# Brain Characterization Using Normalized Quantitative Magnetic Resonance Imaging

**DOI:** 10.1371/journal.pone.0070864

**Published:** 2013-08-05

**Authors:** Jan B. M. Warntjes, Maria Engström, Anders Tisell, Peter Lundberg

**Affiliations:** 1 Center for Medical Image Science and Visualization, CMIV, Linköping University, Linköping, Sweden; 2 Clinical Physiology, Department of Medical and Health Sciences, Linköping University, Department of Clinical Physiology, UHL, County Council of Östergötland, Linköping, Sweden; 3 Radiology, Department of Medical and Health Sciences, Linköping University, Linköping, Sweden; 4 Radiation Physics, Department of Medical and Health Sciences, Linköping University, Department of Radiation Physics, UHL, County Council of Östergötland, Linköping, Sweden; University of Jaén, Spain

## Abstract

**Objectives:**

To present a method for generating reference maps of typical brain characteristics of groups of subjects using a novel combination of rapid quantitative Magnetic Resonance Imaging (qMRI) and brain normalization. The reference maps can be used to detect significant tissue differences in patients, both locally and globally.

**Materials and Methods:**

A rapid qMRI method was used to obtain the longitudinal relaxation rate (R_1_), the transverse relaxation rate (R_2_) and the proton density (PD). These three tissue properties were measured in the brains of 32 healthy subjects and in one patient diagnosed with Multiple Sclerosis (MS). The maps were normalized to a standard brain template using a linear affine registration. The differences of the mean value ofR_1_, R_2_ and PD of 31 healthy subjects in comparison to the oldest healthy subject and in comparison to an MS patient were calculated. Larger anatomical structures were characterized using a standard atlas. The vector sum of the normalized differences was used to show significant tissue differences.

**Results:**

The coefficient of variation of the reference maps was high at the edges of the brain and the ventricles, moderate in the cortical grey matter and low in white matter and the deep grey matter structures. The elderly subject mainly showed significantly lower R_1_ and R_2_ and higher PD values along all sulci. The MS patient showed significantly lower R_1_ and R_2_ and higher PD values at the edges of the ventricular system as well as throughout the periventricular white matter, at the internal and external capsules and at each of the MS lesions.

**Conclusion:**

Brain normalization of rapid qMRI is a promising new method to generate reference maps of typical brain characteristics and to automatically detect deviating tissue properties in the brain.

## Introduction

Magnetic Resonance Imaging (MRI) is a very sensitive method for detecting focal changes in the brain. Using the common conventional imaging methods local tissue deviations are highlighted as darker or lighter areas depending on the choice of scanner parameters. Generally, however, no information is obtained on the absolute differences of the observed changes. This precludes objective measures that can determine the significance of deviating tissue properties. Particularly difficult is to detect diffuse changes throughout the brain since a slight change in signal intensity over a larger area may also be due to, for example, coil sensitivity differences or B_1_ field inhomogeneity. A direct comparison of changes across a group of subjects is virtually impossible, not only due to the differences in size and shape of the individual subjects, but also because the absolute image intensity in conventional MRI varies between subjects and examinations and thus cannot directly be compared.

Multi-parametric quantitative MRI (qMRI) can be used as a solution for objective measures of tissue properties. By qMRI, the physical properties that constitute an MR image, *i.e*. the longitudinal relaxation rate (R_1_), the transverse relaxation rate (R_2_), and the proton density (PD), are directly measured. The qMRI parameters provide an absolute scaling, independent of the MR scanner settings and imperfections [1]. Pathological processes such as axonal damage, gliosis, inflammation and edema are characterized by changes in relaxation behaviour and water content [2]–[6] and can therefore be detected as absolute differences in R_1_, R_2_ and PD values.

In order to generate reference maps of typical brain characteristics for a group, the quantitative maps of all subjects can be transformed into a standard stereotactic space. Spatial normalization to a common brain template [7] is a standard procedure, which has been used in many other applications, for example in studies on white and grey matter reduction in psychiatric and neurological disorders, *e.g.* Alzheimer’s disease and Multiple Sclerosis (MS) [8]–[10]. Another example is functional MRI (fMRI), where spatial normalization allows statistical comparisons of the blood oxygen level dependent (BOLD) time series in the participants during performance of cognitive tasks or during sensory stimuli [11]–[13]. In spatial normalization, the sum of the squared differences between the standard template and the images of individual subjects is minimized by spatial deformations and transformations of the intensity values in each image voxel. This technique is completely automatic and is therefore not sensitive to different experience and skill of the individual examiners. An additional advantage of normalization is that it enables the use of standardized atlases to define the various brain structures. By using this approach on qMRI, characteristic brain maps for an entire group of subjects can be generated, where for each voxel throughout the brain the average and standard deviation of the tissue properties are calculated. In previous work on normalized qMRI, various quantification methods were separately acquired and used for monitoring changes with aging or with diseases such as epilepsy and Multiple Sclerosis [14]–[20]. In this work a qMRI method was used that simultaneously measures all three MR tissue parameters R_1_, R_2_ and PD in a single acquisition within a clinically acceptable time, thereby providing both a rapid and objective method for tissue characterization.

There are two main applications of this technique: firstly, a single patient can be compared to a healthy reference group and specific deviations in the patient’s tissue values can be determined. Since the normal variation of the group is known, the significance of the individual differences can be calculated. This application may form a basis for objective pathology detection without user interaction. Secondly, two or more groups can be compared with each other, such that the individual (focal) changes average out, whereas common, more diffuse differences between brain tissue properties become emphasized. Depending on the size of the group, a high statistical power can be achieved. For both approaches atlas-based definitions can be used for the various brain structures, in order to avoid a subjective drawing of an area of interest.

The aim of this work was to propose a method of generating maps of typical brain characteristics in groups of healthy subjects and patients. To demonstrate the method, cerebral R_1_, R_2_ and PD maps were measured by rapid qMRI in a group of healthy subjects and normalized to a standard brain template. A standard brain atlas was used to calculate the mean qMRI values in a number of regions of interest. As an example of the usability of the method towards automatic pathology detection, the tissue characteristics of an elderly subject and a patient diagnosed with MS were compared to the reference values of the healthy group.

## Methods

### Ethics Statement

The study was approved by the Regional Ethics Committee in Linköping (Dnr. M88-07) and written informed consent was obtained from all participants.

### Workflow

**Figure 1:**
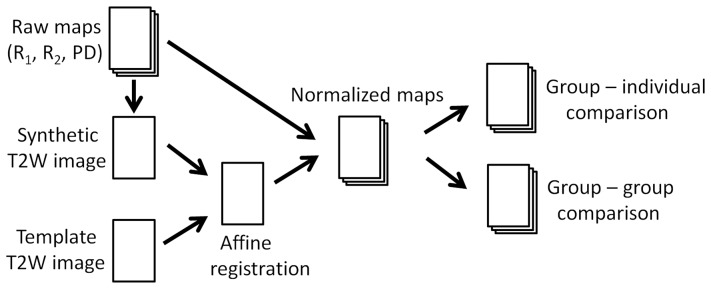
Schematic overview of the method. Magnetic Resonance quantification maps of R_1_ and R_2_ relaxation rates and proton density (PD) are used to synthesize a T2-weighted image. This image is normalized against a standard template T2-weighted image using affine registration. The same transformation is used to normalize the R_1_, R_2_ and PD maps. The individual quantitative maps from a group of subjects are averaged to obtain reference maps of typical cerebral tissue parameters. Such reference maps can either be compared to an individual brain or with another group.

The workflow of the proposed method is schematically depicted in Fig. 1. The measured R_1_, R_2_ and PD maps were used to generate a T2-weighted image using the approach of synthetic MRI [21], [22]. The synthetic T2-weighted image was normalized to a standard T2-weighted brain template using affine transformation. The same transformation was used to normalize the separate maps. The normalized R_1_, R_2_ and PD maps were averaged to obtain mean values and standard deviations of the complete group. Finally, the differences between an individual and a reference group, or between two groups, can be determined. For a Gaussian distribution a significant (*p*<0.05) deviation of an individual compared with the mean of a reference group is found at a difference of, at minimum, 1.96 times the standard deviation. This threshold is generally somewhat higher due to the finite size of the reference group. For a comparison between two groups a two-sided t-test with assumed unequal variances is more appropriate, assuming that the measurement values for both groups have a normal distribution in R_1_, R_2_ and PD.

### Subjects

A group of 32 healthy subjects was examined using qMRI. Of these, 31 were used for the normalized reference group (14 male, 17 female, mean age 45±11 years, range 26–67 years). The remaining healthy subject, a female aged 72 years, was used as one example for the technique. The subjects were regarded as healthy based on an interview on disease history. Additionally, one patient (female, aged 40 years), diagnosed with Clinically Definite Multiple Sclerosis (Relapsing Remitting, disease duration 8 years, Expanded Disability Status Scale EDSS = 3.5) was used as a second example to demonstrate the usability of the method. All data was stored in the hospital’s research PACS.

### Scanning Protocol

For quantification the QRAPMASTER (also ‘qMap’) sequence [23] was used. This is a multi spin-echo saturation recovery sequence with 4 saturation delays and 5 echoes. The saturation delays were at 100, 400, 1380 and 2860 ms with a repetition time (TR) of 2950 ms. The echo time (TE) was set to 14, 28, 42, 56 and 70 ms. Hence each acquisition leads to a matrix of 4×5 = 20 images per slice. The in-plane resolution was 1×1 mm^2^ over a field of view (FOV) of 210 mm; 30 axial slices of 4 mm thickness (no gap) were collected in a scan time of 8∶02 minutes. In addition,n T2-weighted (TR/TE = 6000/100 ms) and FLAIR (TR/TE/TI = 6000/120/2000 ms) images were acquired with the same FOV and number of slices in order to compare qMRI data with conventional imaging. The MR-scanner was an Achieva 1.5 T (Philips Healthcare, Best, The Netherlands).

### Image Post-Processing

The raw image data were analyzed with the SyMRI Brain Studio software (SyntheticMR AB, Linköping, Sweden) to retrieve the R_1_, R_2_ and PD maps as described previously [23]. Using the same software the Brain Parenchymal Fraction (BPF), the total brain volume divided by the intracranial volume, was automatically measured. Additionally the lateral ventricles were manually segmented to obtain the Lateral Ventricle Fraction (LVF), defined as the total lateral ventricle volume divided by the intracranial volume.

Based the R_1_, R_2_ and PD maps a stack of T2-weighted images was synthesized using an TE = 100 ms and a TR = 4500 ms. These images were used as source images to calculate the transformation matrix to a standard stereotactic space in Montreal Neurological Institute (MNI) coordinates using SPM8 (Wellcome Department of Imaging Neuroscience, University College, London, UK). As a first step in this normalization process, the synthetic T2-weighted images were smoothed with an 8 mm Gaussian kernel to reduce the individual anatomical detail. This is required since the source and the MNI template should have approximately the same degree of smoothing. In this study we used the standard T2-weighted template available in SPM8. The synthetic T2-weighted images were transformed to match the size and position of the template by a 12-parameter (translation, rotation, shear, zoom) affine regularization. The same transformation matrix was then applied to the R_1_, R_2_ and PD maps. The resulting data was re-gridded to 2×2×2 mm^3^ to obtain an isotropic dataset. Mean values of R_1_, R_2_ and PD, standard deviations σ(R_1_), σ(R_2_) and σ(PD) and coefficients of variation (CoV = σ/mean) for all voxels in the brain were calculated.

In order to characterize different structures in the brain, we used the Wake Forrest University (WFU) PickAtlas [24] to define regions of interest (ROI). Six ROIs were predefined using the Automated Anatomical Labeling (AAL): the lateral ventricles, insula, the cingulate cortex, caudate nucleus, putamen, thalamus and five ROIs using the Talairach Daemon (TD): midbrain, pons and corpus callosum as well as regions representing whole brain grey matter (GM) and white matter (WM) Four ROIs were created by intersecting the WM ROI with corresponding cerebral lobes, thus obtaining frontal, parietal, occipital and sub-lobar WM. Mean value and standard deviation (SD) for R_1_, R_2_ and PD were calculated for all 15 predefined ROIs by taking the average R_1_, R_2_ and PD values within each individual ROI for the healthy group. Linear regression was performed on these values as a function of subject age.

To determine significant tissue deviations of the elderly subject and the MS patient, the differences between the quantitative tissue characteristics of the subject and the corresponding mean values of the reference group, ΔR_1_ = R_1,s_ – R_1,mean_, ΔR_2_ = R_2,s_ – R_2,mean_ and ΔPD = PD_s_ – PD_mean_, were normalized with the standard deviation σ, obtaining ΔR_1_/σ(R_1_), ΔR_2_/σ(R_2_) and ΔPD/σ(PD). Only significant differences were displayed by showing all data that was higher than a threshold of 2.04, corresponding to p = 0.05 for a reference group of 31 subjects. In order to combine all three qMRI maps the magnitude of the normalized vector sum S was calculated, defined as:
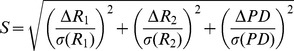
(1)


In the visualization of the combined map all voxels that exceeded a value of 5 (corresponding to p<0.000001) were indicated with a color.

## Results

**Figure 2:**
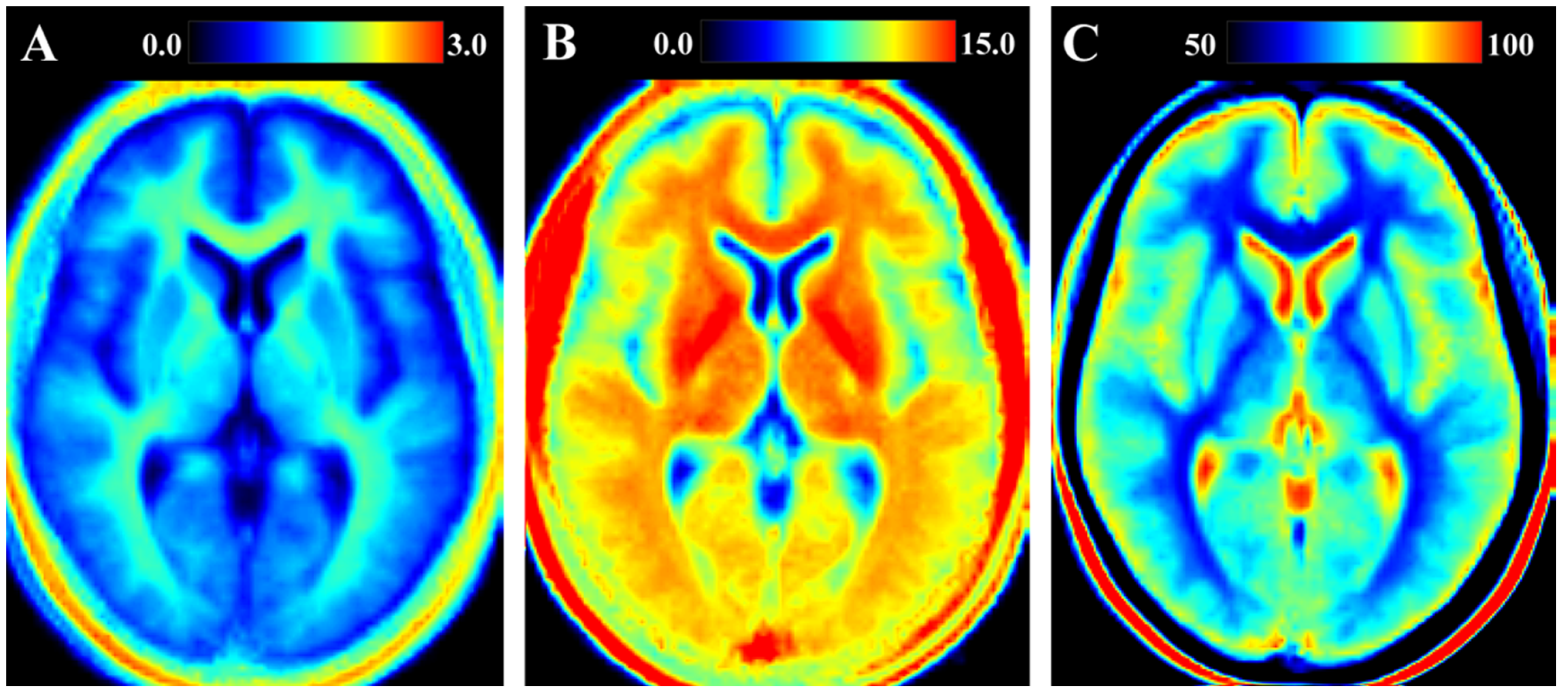
Example of the normalized qMRI reference maps in a slice through the brain. The figure shows the reference maps obtained from images pooled across a group of 31 healthy subjects with **A**: R_1_ relaxation rate on a scale 0–3 s^−1^, **B**: R_2_ relaxation rate on a scale 0–15 s^−1^ and **C**: proton density on a scale 50–100%, where 100% corresponds to pure water at 37°C.

**Figure 3:**
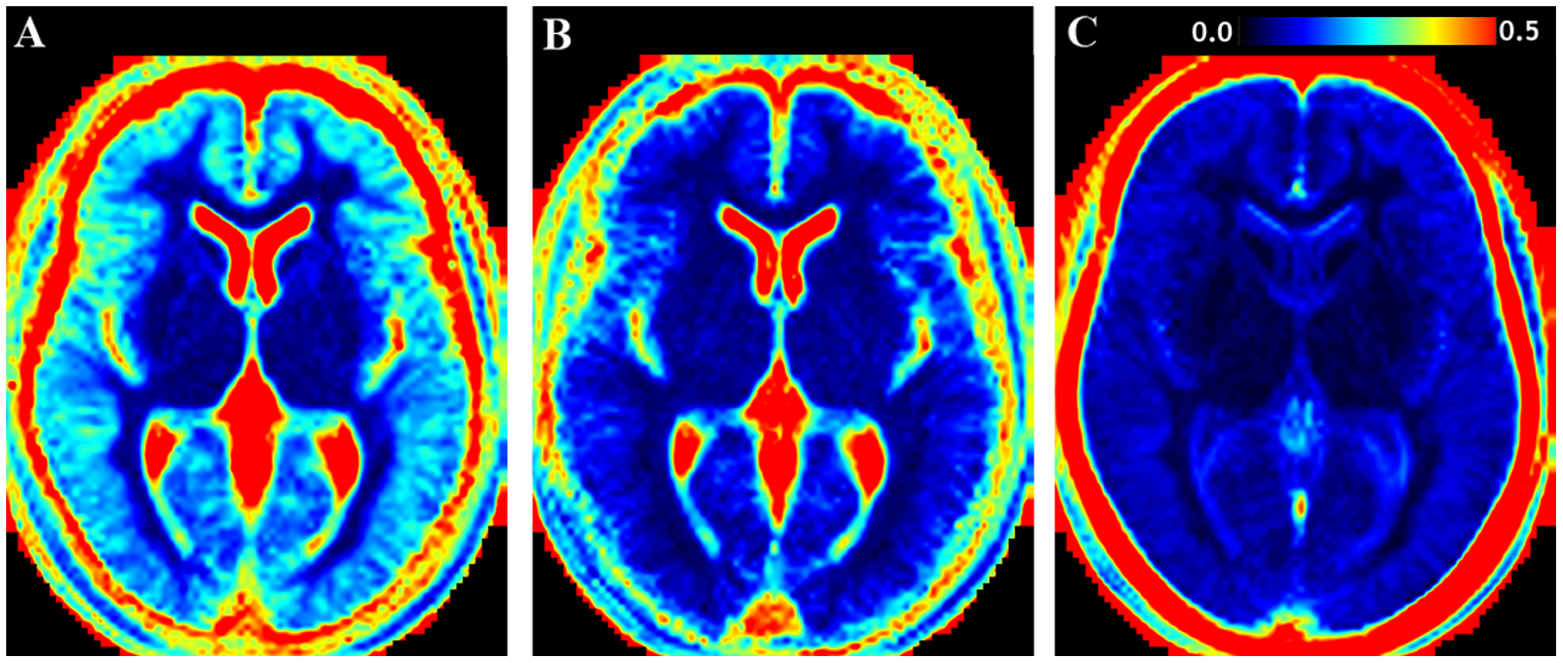
The coefficient of variation (CoV) of the reference maps. The figure shows CoV in the same slice as visualized in Fig. 2, with **A:** CoV map of R_1_, **B:** CoV map of R_2_ and **C:** CoV map of PD. The same scaling of [0–0.5] was used in all CoV maps.

In Fig. 2 one axial slice of the normalized, mean R_1_, R_2_ and PD maps of the group of healthy subjects is shown. Clear differences in R_1_, R_2_ and PD values between the various brain structures are visible. Predefined, atlas-based ROIs that characterize the typical values of the healthy group are listed in Table 1. Also in Table 1 the change in qMRI values per year of subject age is displayed. Significant changes (written in bold face) were observed in all three qMRI parameters in the WM ROIs and in the caudate nucleus. In addition, significant correlation with age was observed for R_1_ in cortical GM and for R_2_ in the thalamus. In Fig. 3 the CoV maps of the three parameters are displayed. The CoV was especially high (>0.4, red in Fig. 3) at the edges of the ventricles and around the brain periphery. For R_1_ the CoV was also relatively high for the cortical grey matter, in the range 0.20–0.25 (light-blue), whereas it was approximately 0.10–0.15 for R_2_ and PD in the same area (dark blue). The CoV in white matter and in the deep grey matter structures such as the head of the caudate nucleus, the putamen and the hypothalamus was generally in the range 0.05–0.10 for R_1_, 0.03–0.07 for R_2_ and 0.02–0.05 for PD. Bands of higher CoV values at tissue interfaces, which might indicate problems in the normalization process, could not be observed.

**Table 1:** The mean value and standard deviation of the R1, R2 and PD values in various regions of interest, defined by using the PickAtlas.

ROI	R_1_	R_2_	PD	R_1_ slope	R_2_ slope	PD slope
	(s^−1^)	(s^−1^)	(%)	(s^−1^/year)	(s^−1^/year)	(%/year)
Lateral ventricles	0.38±0.32	3.89±2.35	94.3±8.8	−0.0075	−0.046	0.17
Insula	0.80±0.07	8.94±0.73	83.1±1.5	−0.0004	−0.006	0.00
Cingulate cortex	0.97±0.05	10.23±0.36	79.9±1.0	−0.0007	−0.005	0.01
Caudate nucleus	1.00±0.15	10.61±1.51	80.3±2.8	−**0.0088**	−**0.091**	**0.15**
Cortical gray matter	1.04±0.09	9.87±0.44	75.1±2.4	**0.0031**	−0.006	−0.03
Pons	1.24±0.07	11.34±0.50	73.5±1.5	−0.0003	0.005	0.00
Putamen	1.25±0.05	13.07±0.42	75.2±1.6	0.0008	0.001	−0.03
Mid brain	1.28±0.05	11.93±0.33	73.2±0.9	−0.0010	−0.007	0.01
Thalamus	1.33±0.07	12.81±0.21	72.5±1.9	−0.0016	−**0.009**	0.04
Occipital white matter	1.33±0.04	11.80±0.26	71.2±1.1	−0.0005	−0.004	0.00
Frontal white matter	1.33±0.06	11.77±0.32	71.1±1.4	−**0.0040**	−**0.016**	**0.08**
Parietal white matter	1.34±0.05	11.76±0.25	71.0±1.2	−0.0002	0.000	0.01
Sublobar white matter	1.37±0.08	11.20±0.46	69.2±1.9	−**0.0043**	−**0.026**	**0.10**
White matter	1.38±0.05	11.79±0.26	70.0±1.0	−**0.0023**	−**0.011**	**0.05**
Corpus callosum	1.39±0.27	10.71±1.58	68.3±5.6	−0.0084	−**0.057**	0.16

Added are the linear regression slopes with subject age. Significant slopes (p<0.05) are displayed in bold face.

**Figure 4:**
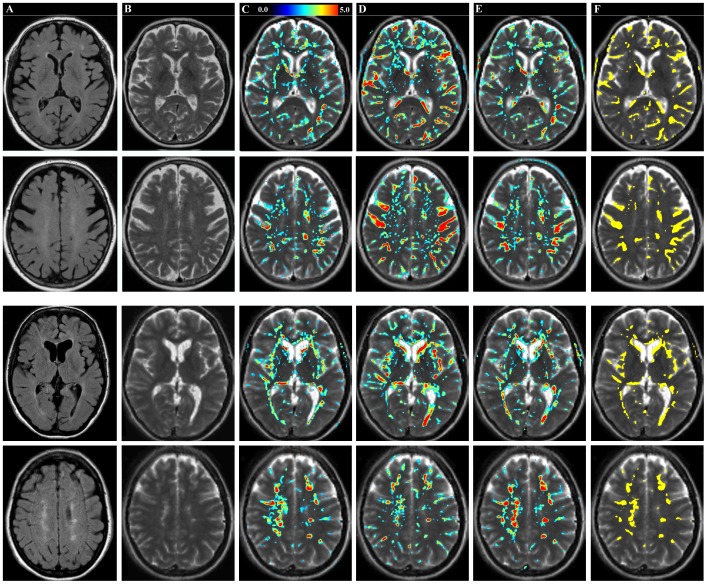
Significant deviation of qMRI tissue properties in an elderly subject and an MS patient. Shown are a FLAIR (**A**) and T2-weighted image (**B**) of two axial slices of the head of an elderly subject (female, 72 years old, top two rows) and an MS patient (female, 40 years old, bottom two rows). The color overlay in **C** corresponds to the normalized difference ΔR_1_/σ(R_1_) between the subject and the group of healthy controls on the same slices. Only values higher than a threshold of 2.04are shown. Synthetic T2-weighted images are calculated using the R_1_, R_2_ and PD maps and used as background images. Similar for ΔR_2_/σ(R_2_) in **D** and ΔPD/σ(PD) in **E**. In **F** the magnitude of the normalized vector sum of ΔR_1_/σ(R_1_), ΔR_2_/σ(R_2_) and ΔPD/σ(PD) is shown where voxels that exceed a threshold of 5 are indicated in yellow.

In Fig. 4 the normalized difference of the R_1_, R_2_ and PD maps of the elderly subject and the MS patient in comparison with the mean reference maps of the healthy group are displayed for two slices through the brain. Note that the scale for R_1_ and R_2_ relaxation is inverted compared to PD because typical differences between the examples and the group of healthy controls consisted of a decrease in R_1_ and R_2_ and an increase in PD. For the elderly subject clear changes were observed along the sulci. A number of focal changes were seen in the periventricular area and minor differences around the ventricles. For the MS patient the largest differences were observed as a rim along the ventricular system. Moreover moderate changes in the MS patient’s R_1_, R_2_ and PD are observed in the periventricular white matter, capsula interna, capsula externa and centrum semiovale. The MS lesions, visible as hyper-intensities in the FLAIR and T2-weighted images in Fig. 4A and B, also show a significant decrease in R_1_, R_2_ and increase in PD compared to healthy white matter at the same location. In the cortical grey matter no significant tissue deviations were observed. In Fig. 4F the vector sum is displayed where only differences are shown that exceed a threshold of 5. This map clearly displays the changes at the sulci of the elderly subject and at the edges of the ventricles, in the capsula externa and at all lesions of the MS patient.

In order to verify the presence of brain atrophy the BPF and LVF were measured for all participants. The mean BPF of the reference group was 90.0±2.5%. Linear regression showed a decrease of BPF of 0.12% (p = 0.003) per year of age. The mean LVF was 1.1±0.5% in the reference group. Linear regression showed an increase of LVF of 0.025% (p<0.001) per year of age. The elderly subject had a BPF of 80.8% and an LVF of 1.7%. This BPF was rather small and significantly deviated from the reference group (−9.2%, p<0.001), although the subject was enlisted as healthy. The LVF difference was not significant (+0.6%, p = 0.2). The MS patient, on the other hand, had a BPF of 84.9% and an LVF of 3.6%. This situation was the reverse in comparison with the elderly subject, the BPF difference was just at the normal significance threshold (−5.1%, p = 0.05) whereas the LVF was significantly higher (+2.5%, p<0.001).

## Discussion

A method is proposed to characterize brain tissue using a combination of rapid quantitative MRI and brain normalization to enable an automatic and objective comparison of tissue properties between groups and individuals. Using this method each voxel throughout the brain lies at the same stereotactic location for all subjects and the quantitative R_1_, R_2_ and PD values can be investigated for differences between groups and individuals. An atlas-based definition ensures an objective description of the various brain structures. The CoV is a good measure for the local sensitivity of the method since low CoV values are only possible when the tissue has low variation in qMRI values and normalization worked well for all subjects. The observed low CoV in white matter and deep grey matter structures of the group of healthy subjects indicates that the method is particularly suitable in these areas. Demonstration of the method on an elderly subject and an MS patient resulted in the observation of significant differences in all three normalized qMRI maps. Combining the maps into a normalized vector map clearly highlighted the subject-specific differences compared to a healthy reference group.

The qMRI values presented in Table 1 were similar to previously reported values with the same quantification method of the brain [23], [25] and close to values obtained by other methods [26]–[28]. The WM values were lower than previously reported values (e.g. T_1_ = 608 ms (R_1_ = 1.64 s^−1^) in [27] and T_1_ = 602 ms (R_1_ = 1.66 s^−1^) in [28]) but this seems mainly to be due to the rather wide region of interest defined in AAL, resulting is a minor overlap with GM areas for our method. A change of qMRI values with subject age was only observed in WM and the caudate nucleus. Although it seems likely that indeed changes in WM are larger than in GM in relation with age it must be noted that the sensitivity of the method was better in WM and the deep GM structures in comparison to cortical GM, which may partially obscure potential changes in cortical GM.

It is not straightforward to attribute the observed differences in the qMRI maps to specific causes regarding tissue microstructure. It seems likely that the changes in the elderly subject at the sulci indicate brain atrophy. This conclusion is supported by the measured low BPF inn the elderly subject. For the MS patient similar effects were observed at the edges of the brain stem and the ventricular system, supported by the large LVF observed in that patient. For results related to potential brain atrophy the decrease of R_1_ and R_2_ relaxation and increase in PD was caused by a partial volume effect where brain tissue was replaced by cerebrospinal fluid, since the total brain parenchymal volume decreased beyond the normal variation in the healthy subjects. The observed changes in the white matter and in the MS lesions, on the other hand, were more likely caused by actual changes in the brain tissue properties, which can be confirmed by the presence of the hyperintensities in the FLAIR and T2-weighted images. The significant deviations in the white matter in the vicinity of the capsula interna and externa, however, were not unambiguous to interpret. These R_1_, R_2_ and PD deviations could be caused by an actual change of the white matter tissue properties or rather a result of imperfect brain normalization. In a recent study by Van Hecke and co-workers [29] diffusion differences between MS patients and controls in this area was observed, indicating white matter aberrations in these regions. It is recognized that objective methods, such as qMRI, are important to quantify differences between patients and the healthy population [30], but more investigation is required to establish the distinction between different causes such as atrophy and actual tissue changes. A potential improvement would be the definition of a geometrical mask to exclude areas with high CSF content or tissue interfaces, to limit the effect of brain atrophy in the qMRI maps.

A limitation of the method is the potential inadequate ability of the geometrical morphing algorithm to successfully register the various brain structures. The natural variation of the size and shape of the brain and of the ventricular system resulted, even for the healthy population, in a high CoV at the interface between the brain and CSF. In addition, the complex shape of the cortical windings is different for each individual, resulting in a higher CoV in the entire cortex. This limits the statistical power in these areas. Resampling the qMRI maps to lower resolution will improve the registration but a trade-off must be made between maintaining anatomical detail and minimizing the CoV on tissue interfaces. It can be expected that these aspects will be particular challenging for patients with *e.g.* severe brain atrophy, hydrocephaly or very large brain pathologies. In future studies we will explore the possibilities for ameliorated normalization by using source images with higher resolution for the calculation of the transformation matrix and by using improved normalization algorithms that, besides a linear affine registration, also apply non-linear procedures.

An area of interest is the choice of the significance threshold. In many applications a significance level of p<0.05 is used, which also was applied in the present study for calculating the tissue maps in Fig. 4C–E. For a normal t-distribution this choice implies that there may be 5% of all pixels in an image that appear to deviate significantly purely by chance. For an image matrix of 256×256 pixels statistically this corresponds to over 3000 pixels. Increasing the threshold to t = 5 decreases this number to less than 0.5 pixels, *i.e*. it is not likely that any pixel in the image will appear to deviate by chance. This threshold was used for the combined quantitative map displayed in Fig. 4F. Further validation is required, however, to define a threshold which constitutes an optimal balance between false positives and false negatives that might be appropriate for clinical applications.

In the future we will aim for automatic pathology detection based on this method. Besides the potential for brain atrophy and MS lesion detection, the proposed method could be applied to objectively measure the volume of brain tumors or the edemic area after stroke [31]–[33]. A significant advantage of the method is that individual segmentation of the brain is no longer required since all brains are mapped to the same standard brain template. Using a standard atlas removes ambiguity concerning the definitions of regions of interest. An inverse transformation of the resulting pathology maps can be co-registered to the conventionally acquired images to indicate the findings and obtain the true volumes of the ROIs. In this work it has been shown that tissue deviations in individual patients can automatically be found. For pathology detection, however, it is required that the method is validated by confirmation of a neuroradiologist. Validation can be combined with a more thorough analysis of the observed qMRI differences using more sophisticated methods such as multivariate or principle component analysis, rather than the example of the vector sum.

The alternative application of the method, to compare two or more groups of subjects to find group-specific features, was not investigated here. Analyzing groups, rather than individuals, will increase the statistical power substantially. However, the method will then be less sensitive to individual, focal changes but will highlight more diffuse differences, common for the whole group. Therefore the proposed method is expected to be suitable for the investigation of diffuse white matter changes such as in Normal Appearing White Matter or Dirty Appearing White Matter (NAWM/DAWM) in MS. It is known that there is only a moderate correlation between the focal changes in the brain of MS patients and clinical disability [34], [35]. We believe that this technique can be applied to shed more insights into the simultaneous diffuse global changes [36]–[39].

In conclusion, we have shown that it is possible to characterize tissue properties for each voxel throughout the brain for a group of subjects using brain normalization of multi-parametric qMRI data. A standard brain atlas can be used to define regions of interest for objective comparison. The method allows automatic determination of significant tissue differences, exemplified with and elderly subject and a patient with MS in comparison to a healthy reference group. The results from this study are promising since the method is automatic, objective and may lead to robust pathology detection.
